# Noninvasive ventilation use and ICU mortality in COVID-19-related acute respiratory failure: a retrospective cohort study

**DOI:** 10.1016/j.clinsp.2026.101159

**Published:** 2026-09-23

**Authors:** Josehélen Martini, Francisco Xavier de Araujo, Gabriela Machado Costa, Camilo Corbellini, Erika Meléndez-Oliva, Eleuterio A. Sánchez-Romero, Rob Sillevis, Juan Nicolás Cuenca-Zaldívar, Viviane Martins Corrêa Boniatti

**Affiliations:** a Hospital Santa Casa de Porto Alegre, Porto Alegre, RS, Brazil; b Universidade Federal de Pelotas (UFPEL), Pelotas, RS, Brazil; c Hospital Nossa Senhora da Conceição, Porto Alegre, RS, Brazil; d Department of Health, LUNEX University of Applied Sciences, Luxembourg; e Grupo de Investigación en Dietética Aplicada, Nutrición y Composición Corporal (DANuC), Department of Optics, Pharmacology and Anatomy, University of Alicante, Alicante, Spain; f Interdisciplinary Research Group on Musculoskeletal Disorders, Madrid, Spain; g Physiotherapy and Orofacial Pain Working Group, Sociedad Española de Disfunción Craneomandibular y Dolor Orofacial (SEDCYDO), Madrid, Spain; h Research Group in Nursing and Health Care, Puerta de Hierro Health Research Institute-Segovia de Arana (IDIPHISA), Majadahonda, Spain; i Department of Rehabilitation Sciences, Florida Gulf Coast University, Fort Myers, FL, USA; j Physical Therapy Unit, Primary Health Care Center “El Abajón”, Las Rozas de Madrid, Spain; k Facultad de Ciencias Biomédicas y de la Salud, Universidad Alfonso X el Sabio (UAX), Avenida de la Universidad 1, Villanueva de la Cañada, Madrid, 28691, Spain

**Keywords:** COVID-19, Acute respiratory failure, Noninvasive ventilation, Intensive care, Mortality

## Abstract

**Background:**

Acute hypoxemic respiratory failure is a severe complication of Coronavirus Disease 2019 (COVID-19) frequently requiring Intensive Care Unit (ICU) admission. The association between Noninvasive Ventilation (NIV) and outcomes in critically ill patients remains uncertain.

**Objective:**

To evaluate the association between NIV use and clinical outcomes in patients with COVID-19-related acute respiratory failure.

**Methods:**

This retrospective cohort study included adults with confirmed COVID-19 admitted to the ICU of a tertiary hospital in Porto Alegre, Brazil, between March and August 2021. Patients were categorized according to two periods with different NIV implementation strategies. Survival was evaluated using Kaplan-Meier analysis, and ICU mortality predictors were assessed using multivariable Cox regression.

**Results:**

A total of 296 patients were included. NIV use increased between periods (2.4%vs. 77.8%; *p* < 0.001). The overall intubation rate was 90.5%, with no significant differences between periods (*p* = 0.139). Patients admitted during the second period had more severe hypoxemia and higher crude ICU mortality (58.5%vs. 43.2%; *p* = 0.013). In the multivariable Cox model, absence of NIV use was associated with a lower adjusted hazard of ICU mortality compared with NIV use (HR = 0.383; 95% CI 0.158–0.929; *p* = 0.034).

**Conclusions:**

NIV use increased markedly but was not associated with lower intubation rates. Patients receiving NIV had higher observed adjusted mortality; however, this association should not be interpreted as evidence of harm from NIV and likely reflects confounding by indication and treatment selection, as NIV was preferentially used in patients with more severe respiratory failure. Prospective studies are needed to clarify the role of NIV in critically ill patients with COVID-19.

## Highlights

• NIV use increased from 2.4% to 77.8% across the two study periods.
• Intubation rates remained high and did not differ significantly between periods.
• Second-period patients had more severe hypoxemia and higher crude ICU mortality.
• Absence of NIV use was associated with a lower adjusted hazard of ICU mortality.
• The association likely reflects confounding by indication, not a causal NIV effect.

## Introduction

Coronavirus Disease 2019 (COVID-19), caused by Severe Acute Respiratory Syndrome Coronavirus-2 (SARS-CoV-2), rapidly became one of the leading causes of hospitalization, critical illness, and mortality worldwide.^1^^,^^2^ Although most infected individuals experience mild or moderate disease, a substantial proportion develop severe pulmonary involvement requiring hospital admission and advanced respiratory support.^2^^,^^3^ Acute hypoxemic Respiratory Failure (ARF) is among the most serious manifestations of severe COVID-19 and represents one of the principal reasons for Intensive Care Unit (ICU) admission.^4^^,^^5^ The pathophysiology of severe COVID-19 involves complex inflammatory, endothelial, and pulmonary vascular alterations that frequently result in profound hypoxemia, respiratory distress, and increased mortality risk.^4^^,^^5^

The management of acute hypoxemic respiratory failure generally follows a progressive escalation of respiratory support. Conventional oxygen therapy is typically used as the initial approach, followed by High-Flow Nasal Oxygen (HFNO) or Noninvasive Ventilation (NIV) when oxygenation remains inadequate.^6^, ^7^, ^8^ In patients with persistent respiratory deterioration, endotracheal intubation and Invasive Mechanical Ventilation (IMV) become necessary to maintain gas exchange and prevent respiratory collapse.^9^ However, IMV has been associated with important complications, including ventilator-associated lung injury, barotrauma, prolonged ICU stay, and adverse clinical outcomes in patients with severe COVID-19.^10^^,^^11^ Consequently, considerable interest has emerged in identifying noninvasive respiratory strategies capable of improving oxygenation while potentially avoiding or delaying invasive ventilation.^6^, ^7^, ^8^

The role of NIV in COVID-19-related ARF evolved substantially throughout the pandemic. During the initial stages, concerns regarding aerosol generation and nosocomial transmission of SARS-CoV-2 prompted many institutions to favor early intubation strategies and limit the use of noninvasive respiratory support.^9^^,^^12^ Subsequent experimental and clinical evidence suggested that when appropriate interfaces and infection-control measures are employed, particle dispersion associated with NIV is limited and comparable to that observed with other oxygen-delivery systems.^10^^,^^11^^,^^13^ These findings, together with the unprecedented demand for respiratory support during pandemic surges, contributed to a progressive increase in the use of noninvasive respiratory strategies in clinical practice.

Accumulating evidence indicates that NIV may improve oxygenation and clinical status in selected patients with COVID-19-related ARF.^13^, ^14^, ^15^, ^16^, ^17^ Several observational studies have reported favorable outcomes associated with NIV or HFNO, including improvements in respiratory parameters and lower intubation rates in specific patient populations.^18^, ^19^ In addition, large multicenter cohorts have demonstrated the feasibility and clinical impact of implementing noninvasive respiratory support strategies in patients with COVID-19 pneumonia, including outside traditional ICU settings.^18^^,^^19^

More recently, randomized studies have provided further insight into the effectiveness of noninvasive respiratory support. The RECOVERY-RS trial demonstrated that Continuous Positive Airway Pressure (CPAP) reduced the composite outcome of tracheal intubation or mortality compared with conventional oxygen therapy in patients with COVID-19-related acute hypoxemic respiratory failure.^20^ Similarly, investigations evaluating different NIV interfaces have suggested that the choice of respiratory support modality may influence clinical outcomes in selected patients.^21^, ^22^

Despite the growing body of evidence supporting noninvasive respiratory strategies, important uncertainties remain regarding their effectiveness in critically ill patients with COVID-19-related ARF. Previous studies have reported heterogeneous findings, likely reflecting differences in patient selection, baseline disease severity, timing of intervention, ventilatory interfaces, and institutional protocols.^6^^,^^13^^,^^14^^,^^17^^,^^18^ Furthermore, most investigations have focused primarily on intubation avoidance, whereas the association between NIV use and survival among critically ill ICU populations remains less clearly defined. The rapid evolution of clinical practice throughout different phases of the pandemic has further contributed to variability in respiratory support strategies and clinical decision-making processes.^9^^,^^11^

However, whether NIV is associated with improved survival among critically ill ICU patients remains uncertain, particularly in real-world settings characterized by changing respiratory support policies throughout the pandemic.

Therefore, the present study aimed to evaluate the association between NIV use and clinically relevant outcomes, including intubation and ICU mortality, among patients admitted to the ICU with COVID-19-related acute respiratory failure. Additionally, we compared outcomes across two institutional periods characterized by different NIV implementation policies to explore the potential impact of changes in respiratory support strategies during the pandemic.

## Methods

### Study design and ethics statement

This retrospective cohort study was conducted in the ICU of Hospital Nossa Senhora da Conceição (HNSC), which is part of the Conceição Hospital Group (Grupo Hospitalar Conceição, GHC) in Porto Alegre, Rio Grande do Sul, Brazil. The study included consecutive patients admitted between March and August 2021 during the Coronavirus Disease 2019 (COVID-19) pandemic.

This study was conducted in accordance with the principles of the Declaration of Helsinki and was approved by the Research Ethics Committee of the Grupo Hospitalar Conceição (GHC), Porto Alegre, Brazil (approval number 4164341). Due to the retrospective nature of the study and the use of anonymized data obtained from electronic medical records, the requirement for informed consent was waived.

For analytical purposes, patients were categorized into two admission periods according to institutional policy regarding the use of NIV during the pandemic: an initial period in which NIV was generally avoided owing to concerns about aerosol generation and a subsequent period after institutional approval of NIV use following emerging evidence on the safety of noninvasive respiratory support.

The design, conduct, and reporting of this study followed the recommendations of the Strengthening the Reporting of Observational Studies in Epidemiology (STROBE) Statement,^23^ which provides methodological guidance for observational studies.

### Participants

Adult patients aged ≥ 18-years who were admitted to the ICU of HNSC with confirmed COVID-19 and Acute Respiratory Failure (ARF) during the study period were included in this study. The diagnosis of COVID-19 was confirmed using Reverse Transcription Polymerase Chain Reaction (RT-PCR) testing or rapid antigen detection testing, according to institutional protocols.

Patients were included if they required escalation of respiratory support due to COVID-19-related ARF during their ICU stay. Clinical and demographic data were retrospectively retrieved from the electronic medical records. Consecutive eligible patients who met these criteria during the study period were included in the analysis. No additional exclusion criteria were applied.

### Data collection procedures

Data collection was conducted by physiotherapists working in the ICU Physiotherapy Service and by residents from the Multiprofessional Health Residency Program of the institution. Prior to the start of the study, all professionals involved in data collection received standardized training to ensure consistency in data recording and adherence to predefined procedures.

Data were collected using a standardized case report form specifically developed for this study (Appendix A) that was adapted from the routine ICU clinical documentation system. Data collection occurred from the time of ICU admission and continued throughout the ICU stay. Ventilatory parameters were recorded at predefined time points after NIV initiation in patients who received NIV.

Baseline demographic and clinical characteristics were obtained from the electronic medical records and included age, sex, comorbidities, date of symptom onset, dates of hospital admission and ICU admission, and laboratory parameters at hospital admission. Laboratory variables included inflammatory and coagulation markers, such as C-reactive protein and D-dimer levels.

### Ventilatory parameters and clinical outcomes

For patients who received NIV, ventilatory parameters were recorded at three predefined time points: 1) On the day of NIV initiation, 2) During the first 24-hours of NIV therapy, and 3) On the final day of NIV use.

The ventilatory parameters recorded at these time points included Inspiratory Positive Airway Pressure (IPAP), Expiratory Positive Airway Pressure (EPAP), and Fraction of inspired Oxygen (FiO_2_). In addition, arterial blood gas analyses were used to calculate the ratio between arterial oxygen partial pressure and fraction of inspired oxygen (PaO_2_/FiO_2_ ratio) as an indicator of oxygenation efficiency and severity of hypoxemic respiratory failure according to the Berlin Definition of ARDS.^24^

The primary outcome was ICU mortality. Secondary outcomes included intubation requirement, duration of invasive mechanical ventilation, tracheostomy, ICU length of stay, hospital length of stay, and in-hospital mortality. NIV failure was defined as the requirement for Orotracheal Intubation (OTI) and subsequent Invasive Mechanical Ventilation (IMV).

### Severity of illness

Disease severity at ICU admission was assessed using the Simplified Acute Physiology Score-3 (SAPS-3), a validated prognostic scoring system designed to estimate mortality risk among critically ill patients.^25^^,^^26^ SAPS-3 incorporates 20 physiological and clinical variables that reflect acute physiological disturbances and pre-existing health conditions.

The score ranges from 0 to 217, with higher values indicating greater disease severity and an increased predicted risk of mortality.^25^^,^^26^ The SAPS-3 requires the assessment of physiological variables within the first hour after ICU admission and is widely used for risk stratification in critical care settings.

### Statistical analysis

All statistical analyses were performed using R (version 4.1.3; R Foundation for Statistical Computing, Vienna, Austria).

A two-sided significance level of *p* < 0.05 was used for all statistical tests.

Qualitative variables are described using absolute frequencies and percentages, whereas quantitative variables are summarized using median and interquartile range.

The distribution of quantitative variables was evaluated using the Kolmogorov-Smirnov test with the Lilliefors correction. Comparisons between the two study periods were conducted using the Mann-Whitney *U test* for quantitative variables and Fisher’s exact test for qualitative variables.

Survival analysis was performed to evaluate mortality outcomes. A Cox proportional hazards regression model was used to identify independent predictors of mortality. Variables were selected a priori according to clinical relevance and previous evidence from critical care literature. These included the study period, use of NIV, Simplified Acute Physiology Score-3, C-reactive protein, D-dimer, and the PaO_2_/FiO_2_ ratio at admission, all of which have previously been associated with disease severity, organ dysfunction, respiratory failure, or mortality in critically ill populations.^24^, ^25^, ^26^, ^27^, ^28^

Multicollinearity among predictors was assessed using the Variance Inflation Factor (VIF), and variables with a VIF greater than 5 were excluded from the model.

The proportional hazards assumption of the Cox model was verified using Schoenfeld residual tests for each individual variable and for the overall model.

Variables for the multivariable Cox model were not selected through automated stepwise procedures to prevent selection bias and overfitting. Instead, a robust two-stage screening process was used. First, 9 clinical covariates were selected a priori based on critical care literature. Second, due to the high percentage of missing data detected (Supplementary Material Table 1), a sensitivity analysis comparing complete-case naïve data (*n* = 86) against multiply imputed datasets (*n* = 296) was conducted to test parameter stability.

For the multiple imputation framework, a Missing at Random (MAR) mechanism was assumed, and *m* = 5 imputed datasets were generated. To prevent statistical bias, the imputation model included all predictors as well as the survival outcomes (the event indicator ‒ number of deaths ‒ and time to event ‒ days in ICU).

The choice of imputation method was conditioned on variable type: Predictive Mean Matching (PMM) was utilized for continuous variables, binary logistic regression for dichotomous variables (period, NIV use, and tracheostomy), and polytomous logistic regression for multi-category covariates (comorbidities).

Imputation convergence was confirmed via visual inspection of Markov Chain Monte Carlo (MCMC) trace plots, which showed well-mixed and stable chains throughout the iterations for all imputed covariates (Supplementary Material Fig. 1).

Finally, multivariate pooled estimates from the imputed framework were compared against the original complete-case analysis. Sensitivity analyses were performed to evaluate parameter stability across complete-case and multiply imputed datasets. The predictors that remained significant across both analyses were considered robust.

## Results

### Study population

A total of 300 patients admitted to the ICU with confirmed COVID-19-related Acute Respiratory Failure (ARF) were screened. After applying the eligibility criteria and excluding patients with missing information in key analytical variables, 296 patients were included in the final analysis. Reasons for exclusion and final cohort allocation are detailed in Fig. 1.

**Fig. 1:**
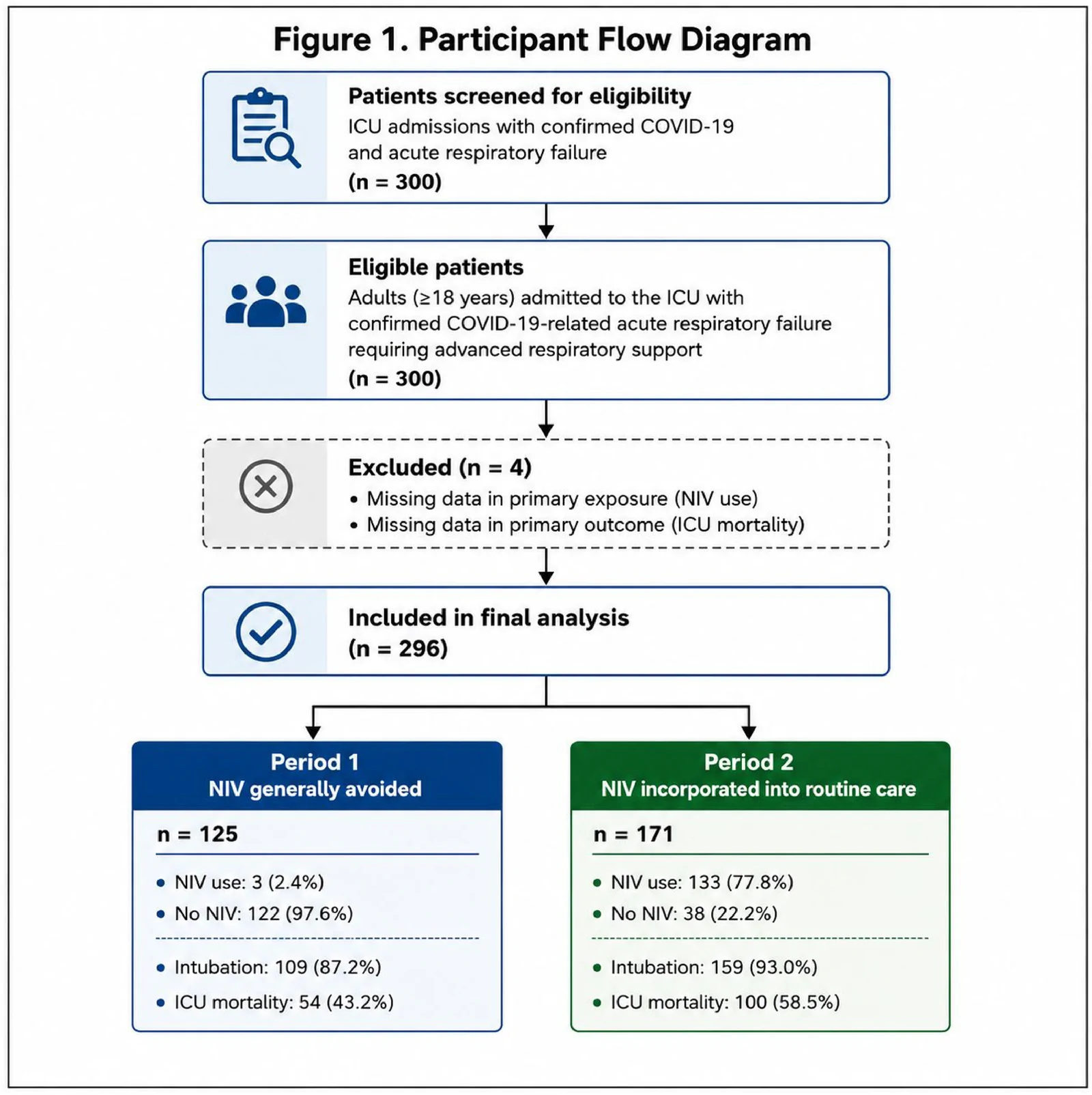
Flow diagram of participant selection and cohort allocation. A total of 300 adult patients admitted to the Intensive Care Unit (ICU) with confirmed COVID-19 ‒ related acute respiratory failure was screened for eligibility. Four patients were excluded because of missing data in key analytical variables, resulting in a final cohort of 296 patients. Participants were subsequently classified according to the institutional period of admission: Period 1, during which Noninvasive Ventilation (NIV) was generally avoided because of concerns regarding aerosol generation (*n* = 125), and Period 2, after institutional approval and routine implementation of NIV (*n* = 171). The number of patients receiving NIV, requiring endotracheal intubation, and experiencing ICU mortality within each period is displayed. ARF, Acute Respiratory Failure; COVID-19, Coronavirus Disease 2019; ICU, Intensive Care Unit; NIV, Noninvasive Ventilation.

Patients were categorized according to their institutional admission periods. The first period included 125 patients and corresponded to the initial phase of the pandemic, when NIV was generally avoided because of concerns regarding aerosol generation. The second period included 171 patients and began after institutional approval of NIV use, following emerging evidence suggesting a low risk of aerosol dispersion when appropriate noninvasive respiratory support interfaces and infection-control measures were used.

### Baseline demographic and clinical characteristics

Baseline demographic and clinical characteristics according to the institutional study period are presented in Table 1.

**Table 1:** Clinical and demographic characteristics of the participants.

		Overall	First period	Second period	^a^*p* Value
*n*		296	125	171	NA
**Socio-demographic characteristics**					
Gender, *n*(%)	Female	140 (47.3)	55 (44.0)	85 (49.7)	0.393
	Male	156 (52.7)	70 (56.0)	86 (50.3)	NA
Age		62.0 [48.0, 69.0]	63.0 [49.0, 71.0]	61.0 [47.5, 68.0]	0.281
Body Mass Index		29.1 [25.6, 32.5]	28.4 [24.2, 32.4]	29.7 [26.3, 32.5]	0.048
Comorbidities, *n*(%)	Cardiovascular	140 (47.3)	56 (44.8)	84 (49.1)	0.056
	Infectious Oncology	17 (5.7)	10 (8.0)	7 (4.1)	NA
	Metabolic	25 (8.4)	10 (8.0)	15 (8.8)	NA
	Others	15 (5.1)	7 (5.6)	8 (4.7)	NA
	Renal	4 (1.4)	1 (0.8)	3 (1.8)	NA
	Respiratory	24 (8.1)	18 (14.4)	6 (3.5)	NA
	Missing data	71 (24.0)	23 (18.4)	48 (28.1)	NA
**Clinical data at admission**					
SAPS-3		62.0 [52.0, 73.0]	64.0 [51.0, 75.0]	61.5 [54.2, 70.0]	0.759
C-Reactive protein (mg/L)		138.8 [78.4, 226.5]	125.8 [76.4, 237.8]	145.9 [79.0, 220.1]	0.635
D-Dimer (μg/L)		1109.0 [687.5, 2479.5]	1108.0 [661.0, 3676.5]	1109.0 [708.0, 1897.0]	0.659
PaO_2_/FiO_2_ ratio (mmHg)		138.0 [92.8, 190.5]	163.0 [122.8, 214.8]	112.0 [80.0, 163.0]	<0.001
**Clinical data in ICU**					
NIV use, *n*(%)	No	160 (54.1)	122 (97.6)	38 (22.2)	<0.001
	Yes	136 (45.9)	3 (2.4)	133 (77.8)	NA
Intubation need, *n*(%)	No	28 (9.5)	16 (12.8)	12 (7.0)	0.139
	Yes	268 (90.5)	109 (87.2)	159 (93.0)	NA
Mechanical ventilation (days)		14.0 [7.0, 23.0]	10.0 [5.0, 18.0]	17.5 [10.0, 25.8]	<0.001
Tracheostomy, *n*(%)	No	255 (86.1)	116 (92.8)	139 (81.3)	0.016
	Yes	40 (13.5)	9 (7.2)	31 (18.1)	NA
	Missing data	1 (0.3)	0 (0.0)	1 (0.6)	NA
ICU death, *n*(%)	No	142 (48.0)	71 (56.8)	71 (41.5)	0.013
	Yes	154 (52.0)	54 (43.2)	100 (58.5)	NA
Days in ICU		19.0 [12.0, 30.0]	15.0 [9.0, 25.0]	22.0 [14.5, 31.0]	<0.001

ICU: Intensive Care Unit; NIV: Non-invasive mechanical ventilation; SAPS-3: Simplified Acute Physiology Score-3. Data expressed with median [interquartile range] or with absolute and relative values (%). ^a^Statistically significant if *p* < 0.05 (shown in red).

The median age of the overall cohort was 62.0 [48.0, 69.0] years. The sex distribution was balanced, with 140 women (47.3%) and 156 men (52.7%). No statistically significant differences were observed between the first and second periods regarding age (63.0 [49.0, 71.0] vs. 61.0 [47.5, 68.0] years; *p* = 0.281) or sex distribution (female sex: 44.0% vs. 49.7%; *p* = 0.393).

Baseline comorbidities were also comparable between periods. Cardiovascular comorbidities were the most frequent comorbidity category, being present in 140 patients (47.3%) overall, with no statistically significant difference between periods (44.8%vs. 49.1%; *p* = 0.056).

Several clinically relevant differences were observed between the periods. Patients admitted during the second period had a higher body mass index than those admitted during the first period (29.7 [26.3, 32.5] vs. 28.4 [24.2, 32.4]; *p* = 0.048). In addition, patients in the second period presented with significantly worse oxygenation at ICU admission, as reflected by a lower PaO_2_/FiO_2_ ratio (112.0 [80.0, 163.0] vs. 163.0 [122.8, 214.8] mmHg; *p* < 0.001). No statistically significant differences were observed between periods for SAPS-3 score, C-reactive protein, or D-dimer levels at admission.

NIV use differed markedly between periods. NIV was used in 3 patients (2.4%) during the first period and in 133 patients (77.8%) during the second period (*p* < 0.001).

### Clinical outcomes during ICU stay

Clinical outcomes observed during ICU admission are also summarized in Table 1.

Overall, 268 patients (90.5%) required orotracheal intubation during their ICU stay. The intubation rate was 87.2% (109/125) in the first period and 93.0% (159/171) in the second period, with no statistically significant difference between periods (*p* = 0.139).

Patients admitted during the second period had a longer duration of invasive mechanical ventilation than those admitted during the first period (17.5 [10.0, 25.8] vs. 10.0 [5.0, 18.0] days; *p* < 0.001). Tracheostomy was also more frequent during the second period (18.1%vs. 7.2%; *p* = 0.016).

The ICU mortality rate was 52.0% in the overall cohort. Mortality was significantly higher during the second period than during the first period (58.5%vs. 43.2%; *p* = 0.013). ICU length of stay was also longer during the second period (22.0 [14.5, 31.0] vs. 15.0 [9.0, 25.0] days; *p* < 0.001).

### Missing data, imputation diagnostics, and model assessment

The proportion of missing data varied across variables included in the analysis. Missing data patterns and percentages for all variables are detailed in Supplementary Table 1. The highest proportion of missingness was observed for SAPS-3 at admission (58.45%), followed by comorbidities (23.99%), PaO_2_/FiO_2_ ratio at admission (21.96%), D-dimer at admission (8.45%), body mass index (6.42%), and C-reactive protein at admission (5.07%). NIV use, age, sex, intubation requirement, ICU mortality, and days in the ICU had no missing data.

Convergence of the multiple imputation procedure was assessed through Markov Chain Monte Carlo trace plots. Visual inspection demonstrated stable and well-mixed chains across iterations for all imputed variables, supporting adequate convergence of the imputation process. The convergence diagnostics are presented in Supplementary Fig. 1.

No predictor exceeded the predefined multicollinearity threshold according to variance inflation factor analysis.

Sensitivity analyses comparing complete-case naïve data with multiply imputed datasets are presented in Supplementary Table 2. The absence of NIV use was the only predictor that remained statistically significant across both analytical frameworks. In the complete-case model, absence of NIV use was associated with a lower hazard of ICU mortality (HR = 0.383; 95% CI 0.158–0.929; *p* = 0.034). This association remained statistically significant after multiple imputations (HR = 0.506; 95% CI 0.299–0.855; *p* = 0.011).

The proportional hazards assumption was evaluated using Schoenfeld residual test. The global test showed no statistically significant violation of the proportional hazard’s assumption (*p* = 0.096). Mechanical ventilation duration was the only variable demonstrating evidence of non-proportionality (*p* = 0.001). Given its clinical relevance and its role as an adjustment variable, it was retained in the multivariable model. Because only one adjustment variable violated the proportional hazards assumption and did not materially alter the main exposure estimate, the model was retained and interpreted cautiously. The complete proportional hazards diagnostics are reported in Supplementary Table 2.

### Multivariable Cox regression analysis for ICU mortality

The results of the multivariable Cox proportional hazards regression model are presented in Table 2.

**Table 2:** Cox model: Mortality in ICU results.

	Hazard ratio (SE)	95% CI	Z (^a^*p V*alue)
Period (Second period)	1.457 (0.469)	(0.581 - 3.653)	Z = 0.803, *p* = 0.422
NIV use (No)	0.383 (0.452)	(0.158 - 0.929)	Z = −2.122, *p* = 0.034
SAPS-3 at admission	1.016 (0.013)	(0.99 - 1.043)	Z = 1.228, *p* = 0.219
C-Reactive protein at admission (mg/L)	0.998 (0.002)	(0.995 - 1.001)	Z = −1.227, *p* = 0.22
D-dimer at admission (μg/L)	1 (0)	(1 - 1)	Z = −0.224, *p* = 0.822
PaO_2_/FiO_2_ ratio at admission (mmHg)	0.995 (0.003)	(0.989 - 1)	Z = −1.855, *p* = 0.064
Body Mass Index	0.973 (0.03)	(0.918 - 1.031)	Z = −0.919, *p* = 0.358
Tracheostomy (Yes)	1.09 (0.648)	(0.306 - 3.884)	Z = 0.132, *p* = 0.895
Mechanical ventilation (days)	0.969 (0.021)	(0.93 - 1.009)	Z = −1.508, *p* = 0.132

Green: Robust variables in sensitivity analysis. ICU: Intensive Care Unit; NIV: Non-invasive mechanical ventilation; SAPS-3: Simplified Acute Physiology Score-3. SE: Standard error; 95% CI: 95% confidence interval. ^a^Statistically significant at *p* < 0.05 (shown in red).

After adjustment for admission period, SAPS-3 score, C-reactive protein, D-dimer, PaO_2_/FiO_2_ ratio at admission, body mass index, tracheostomy, and duration of mechanical ventilation, absence of NIV use was statistically associated with a lower adjusted hazard of ICU mortality compared with NIV use (HR = 0.383; 95% CI 0.158–0.929; *Z* = −2.122; *p* = 0.034). This estimate corresponds to a 61.7% lower adjusted hazard of ICU mortality associated with absence of NIV exposure in the fitted model.

Admission period was not independently associated with ICU mortality after adjustment for baseline severity and oxygenation parameters (HR = 1.457; 95% CI 0.581–3.653; *Z* = 0.803; *p* = 0.422). Likewise, SAPS-3 score, C-reactive protein, D-dimer, PaO_2_/FiO_2_ ratio, body mass index, tracheostomy, and duration of mechanical ventilation were not statistically significant predictors in the final multivariable model.

### Kaplan-Meier survival analysis

Kaplan-Meier survival analysis demonstrated a marked decline in survival probability during the first 30-days after ICU admission, reflecting the high severity of COVID-19-related acute respiratory failure in this ICU cohort.

The unadjusted Kaplan–Meier survival curves according to admission period are presented in Fig. 2. Survival curves initially followed a similar trajectory and crossed during follow-up. The log-rank test showed no statistically significant difference in crude survival between the first and second periods (*p* = 0.99), despite the higher crude ICU mortality observed during the second period in Table 1. These findings suggest that unadjusted survival patterns by period should be interpreted cautiously in the context of the baseline severity differences observed between study periods.

**Fig. 2:**
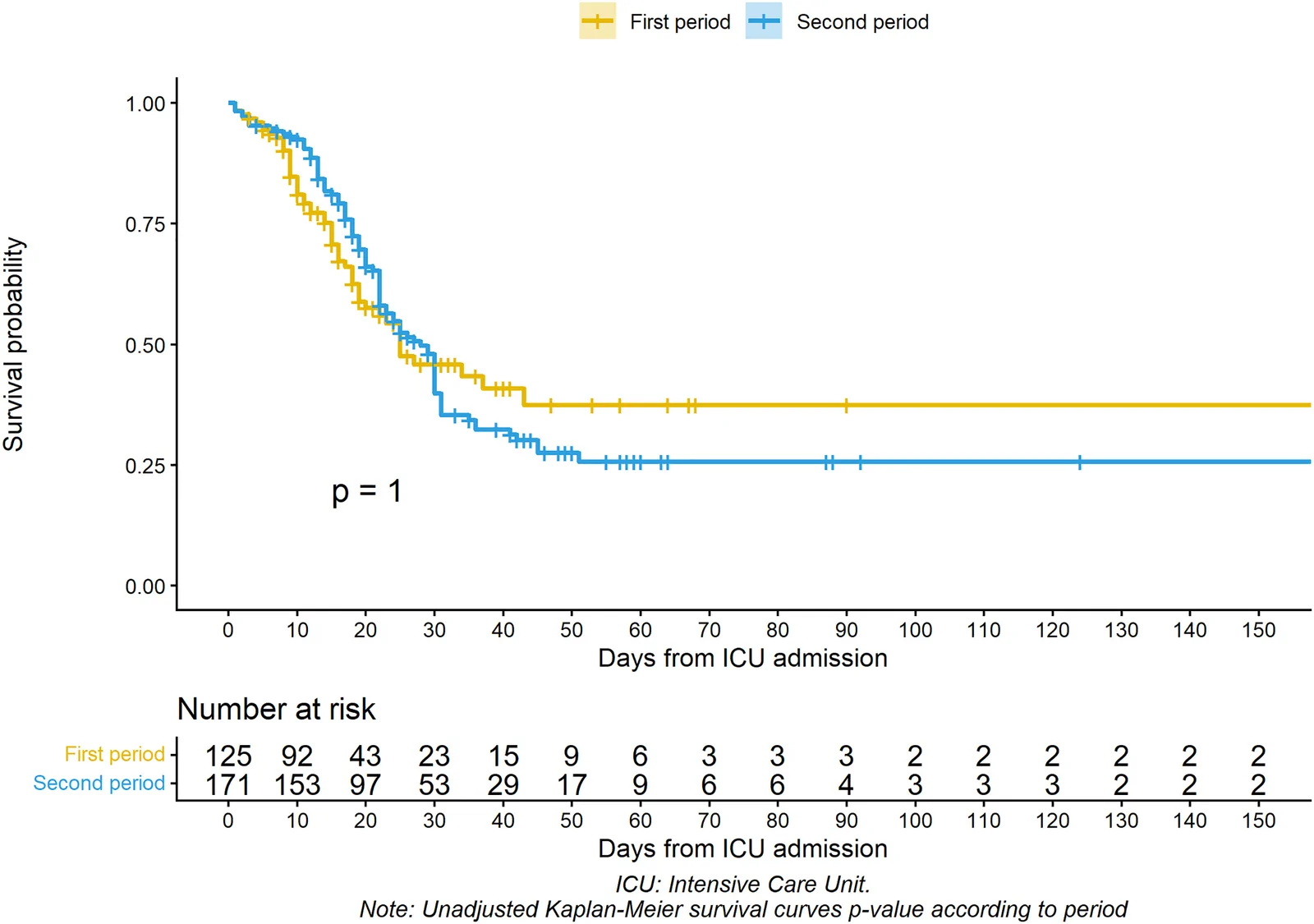
Kaplan-Meier survival curves comparing ICU survival according to institutional admission period. The first period corresponds to the phase during which NIV was generally avoided because of concerns regarding aerosol generation, whereas the second period corresponds to the phase following institutional approval and routine implementation of NIV. Curves represent unadjusted Kaplan-Meier estimates, and the displayed *p*-value corresponds to the unadjusted log-rank test. ICU, Intensive Care Unit; NIV, Noninvasive Ventilation.

The unadjusted Kaplan-Meier survival curves according to NIV exposure are presented in Fig. 3. Patients who did not receive NIV showed a higher crude survival probability than those treated with NIV. However, the between-group difference did not reach statistical significance in the unadjusted log-rank analysis (*p* = 0.20). Although the crude log-rank comparison did not reach statistical significance, the direction of the survival curves was consistent with the multivariable Cox regression model, which identified absence of NIV use as independently associated with a lower hazard of ICU mortality after adjustment for baseline covariates.

**Fig. 3:**
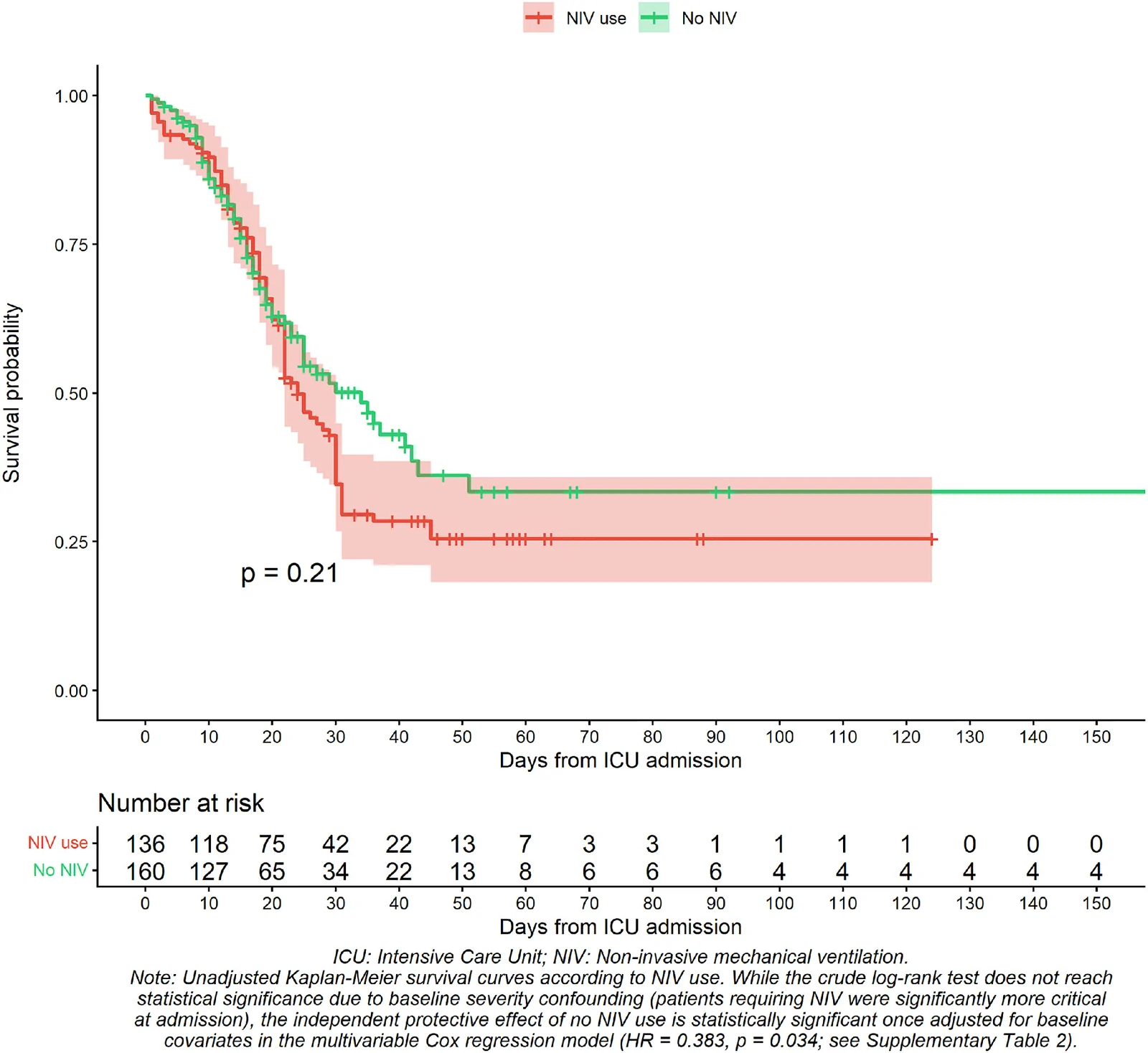
Kaplan-Meier survival curves comparing ICU survival according to noninvasive ventilation exposure. The curves represent unadjusted Kaplan-Meier estimates according to NIV exposure. The displayed *p*-value corresponds to an unadjusted log-rank test. Although the crude log-rank comparison did not reach statistical significance, absence of NIV use remained statistically associated with a lower hazard of ICU mortality after adjustment for measured covariates in the Cox proportional hazards model. ICU, Intensive Care Unit; NIV, Noninvasive Ventilation.

## Discussion

### Principal findings

This retrospective cohort study evaluated the association between NIV use and ICU outcomes among critically ill patients with COVID-19-related acute respiratory failure during two institutional periods characterized by different NIV policies. The main findings were as follows: first, NIV use increased markedly after institutional approval of noninvasive respiratory support; second, patients admitted during the second period presented with significantly worse oxygenation at ICU admission and experienced longer invasive mechanical ventilation, higher tracheostomy rates, longer ICU stay, and higher crude ICU mortality; third, the overall intubation rate remained extremely high and did not differ significantly between periods; and fourth, after multivariable adjustment, absence of NIV use was statistically associated with a lower adjusted hazard of ICU mortality compared with NIV use, whereas admission period was not independently associated with mortality.

These findings should be interpreted within the clinical and methodological complexity of a real-world ICU cohort during the COVID-19 pandemic. The observed association between NIV use and higher adjusted ICU mortality does not establish that NIV caused worse outcomes. Rather, it likely reflects the fact that NIV was preferentially used in patients with more severe respiratory impairment, greater oxygen requirements, and higher risk of clinical deterioration. This interpretation is particularly relevant given the substantial baseline imbalance in oxygenation between periods, the non-random clinical allocation of respiratory support, and the use of NIV as a treatment-escalation strategy in patients with more severe respiratory failure.

### Interpretation of the association between NIV use and ICU mortality

The finding that patients who did not receive NIV had a lower adjusted hazard of ICU mortality must be interpreted cautiously. In observational critical care research, respiratory support strategies are strongly influenced by clinician judgment, baseline disease severity, perceived reversibility, ventilatory demand, oxygenation impairment, neurological status, hemodynamic stability, and institutional protocols. Therefore, NIV exposure may function not only as a treatment variable but also as a marker of clinical severity and therapeutic escalation.

This phenomenon is consistent with confounding by indication, whereby patients receiving a given intervention are systematically different from those who do not receive it. In the present cohort, NIV was implemented much more frequently during the second period, a period also characterized by substantially worse oxygenation at ICU admission. Although the multivariable model adjusted for measured severity indicators, including SAPS-3 and PaO_2_/FiO_2_ ratio, residual confounding remains highly plausible. Variables such as respiratory rate, work of breathing, respiratory drive, vasopressor requirement, awake proning, NIV duration, interface type, tolerance, adherence, timing of NIV initiation, and criteria for intubation were not available for adjustment. Consequently, the association between NIV use and mortality should be considered an adjusted statistical association rather than evidence of a direct harmful effect of NIV.

In the clinical context of this cohort, NIV was preferentially deployed as an escalation strategy in patients with more severe respiratory failure and greater oxygenation impairment. Accordingly, NIV exposure is likely to capture not only receipt of a respiratory intervention but also the clinical selection process that identified patients at higher underlying risk.

The absence of an independent association between admission period and ICU mortality after adjustment is compatible with an important contribution of baseline severity and case-mix differences to the observed crude outcome differences. Although crude mortality was higher during the second period, this temporal effect disappeared in the adjusted Cox model, suggesting that the apparent worsening of outcomes over time was more likely explained by differences in patient severity than by the institutional period itself. This is clinically relevant because the second period combined two simultaneous phenomena: broader use of NIV and admission of patients with more severe hypoxemic respiratory failure. Consequently, NIV exposure should be interpreted primarily as a marker of disease severity and treatment escalation rather than as evidence of a causal effect on mortality.

### Comparison with previous observational evidence

Previous observational studies evaluating NIV in COVID-19-related acute hypoxemic respiratory failure have reported heterogeneous findings. Some cohorts suggested that NIV or other forms of noninvasive respiratory support may improve oxygenation and reduce the need for intubation in selected patients, whereas others reported high failure rates and poor outcomes among patients who ultimately required invasive mechanical ventilation.^12^, ^13^, ^14^, ^15^, ^16^, ^17^, ^18^, ^19^ This variability likely reflects major differences in patient selection, disease severity, ventilatory interface, monitoring intensity, ICU capacity, treatment protocols, and thresholds for intubation. The heterogeneity across observational studies may also reflect differences in ARDS severity, ICU admission criteria, and local thresholds for escalation to invasive mechanical ventilation.^6^^,^^24^

For example, studies conducted during the early pandemic frequently reported worse outcomes among patients requiring NIV, particularly when NIV failure was followed by invasive mechanical ventilation.^12^^,^^17^ Conversely, other cohorts suggested that noninvasive respiratory support could be feasible and clinically useful when applied to carefully selected patients and supported by close monitoring.^8^^,^^13^^,^^16^^,^^19^ The present findings add to this heterogeneous body of literature by illustrating how outcomes observed among NIV-treated patients in routine ICU practice may be strongly influenced by patient selection, baseline respiratory severity, and treatment-escalation pathways. Instead, its clinical effect appears highly dependent on patient phenotype, timing, monitoring, and escalation strategy.

The very high intubation rate observed in this cohort further suggests that NIV frequently functioned as a bridge or escalation strategy rather than a definitive alternative to invasive ventilation. This is important because the effectiveness of NIV in acute hypoxemic respiratory failure is closely linked to early identification of treatment failure. When most patients eventually require intubation, the clinical question shifts from whether NIV prevents intubation to whether NIV is being used in the right patients, at the right time, and with sufficiently strict criteria for escalation.

### Comparison with randomized evidence

Randomized evidence provides a more balanced framework for interpreting these findings. In the RECOVERY-RS trial, CPAP reduced the composite outcome of tracheal intubation or mortality compared with conventional oxygen therapy in patients with COVID-19-related acute hypoxemic respiratory failure.^20^ However, the benefit was mainly driven by reduced intubation rather than a clearly demonstrated independent mortality reduction. Similarly, the HENIVOT trial found no significant difference in respiratory support-free days between helmet NIV and high-flow nasal oxygen, although helmet NIV was associated with a lower rate of endotracheal intubation.^22^

These trials suggest that NIV or CPAP may reduce intubation in selected patients under controlled conditions. However, they do not support a simple assumption that NIV consistently improves survival across all critically ill ICU populations. The present study differs from randomized trials because NIV was not assigned by protocol but was introduced as part of evolving real-world institutional practice during a pandemic. Therefore, the observed association between NIV exposure and higher adjusted mortality likely reflects differences in treatment allocation and baseline severity rather than a contradiction of randomized evidence.

### Timing of intubation, NIV failure, and P-SILI

The high intubation rate observed in the present cohort makes the timing of escalation from NIV to invasive mechanical ventilation particularly relevant. The concern that delayed intubation after NIV failure may worsen outcomes has been widely debated in COVID-19 and non-COVID acute hypoxemic respiratory failure. Papoutsi et al. synthesized data from 12 non-randomized cohort studies including nearly 9000 critically ill COVID-19 patients and found no statistically detectable difference in mortality between early and late intubation strategies.^29^ Importantly, their sensitivity analysis comparing patients intubated with versus without a prior trial of HFNO or NIV also did not demonstrate a clear mortality difference.^29^ These findings support an individualized approach rather than universal early intubation or universal prolonged noninvasive support.

The concept of Patient Self-Inflicted Lung Injury (P-SILI) provides one possible physiological explanation for why prolonged spontaneous breathing during severe hypoxemic respiratory failure could theoretically worsen lung injury. Brochard et al. proposed that excessive inspiratory effort may generate large transpulmonary pressure swings, high tidal volumes, pendelluft phenomena, and increased pulmonary vascular stress, potentially amplifying lung injury in susceptible patients.^30^ This mechanism is biologically plausible and may be especially relevant when patients with severe respiratory drive remain non-intubated for prolonged periods.

However, P-SILI remains a controversial construct in COVID-19. Tobin et al. cautioned that direct human evidence proving clinically meaningful P-SILI is limited and argued that this hypothesis should not be used as a standalone justification for routine early intubation.^31^ This balanced view is particularly applicable to the present cohort. Because respiratory drive, tidal volume during NIV, transpulmonary pressure, esophageal pressure, work of breathing, and NIV duration before intubation were not available, our data cannot determine whether P-SILI contributed to mortality. The results therefore support cautious clinical interpretation: NIV failure and delayed escalation may be relevant mechanisms, but they cannot be confirmed in this retrospective dataset. Therefore, any potential contribution of delayed intubation or P-SILI to the observed mortality differences remains speculative and cannot be established from the present dataset.

### Prognostic markers and risk stratification

Although absence of NIV use was the only predictor that remained consistently significant across complete-case and multiple-imputation analyses, the inclusion of established severity and inflammatory markers in the multivariable model remains clinically relevant. SAPS-3 has been extensively validated as a prognostic tool in critically ill populations and continues to demonstrate good performance in patients with severe respiratory failure and COVID-19.^25^^,^^26^ The markedly reduced PaO_2_/FiO_2_ values observed across both study periods indicate that a substantial proportion of patients fulfilled criteria for moderate-to-severe ARDS according to the Berlin Definition, reinforcing the overall severity of respiratory dysfunction in this cohort.^24^

Similarly, systemic inflammation represents a central component of severe COVID-19 pathophysiology. Elevated C-reactive protein levels have previously been associated with organ dysfunction, disease progression, and mortality in critically ill patients, supporting their inclusion as markers of disease severity and adverse prognosis.^27^

Coagulation abnormalities also play an important role in the development of severe COVID-19. D-dimer elevation has consistently been associated with thrombotic complications, microvascular dysfunction, and increased mortality risk. Recent evidence suggests that composite biomarkers integrating inflammatory, nutritional, and coagulation pathways, such as the albumin-to-D-dimer ratio, may provide improved prognostic information in mechanically ventilated ICU populations.^28^ Although these biomarkers were not independently associated with mortality in the final model, they remain clinically relevant indicators of disease severity and should continue to be explored in future prognostic studies.

### Clinical implications

The present findings should not be interpreted as evidence that NIV is ineffective or harmful in critically ill patients with COVID-19-related acute respiratory failure. Rather, they illustrate the difficulty of evaluating respiratory support strategies in real-world settings in which treatment allocation is strongly influenced by disease severity. In this cohort, NIV was preferentially used as a treatment-escalation strategy in patients with more severe respiratory failure, and its markedly increased use was not accompanied by lower observed intubation rates. Therefore, the principal clinical implication is not avoidance of NIV, but the importance of appropriate patient selection, close physiological monitoring, and timely recognition of treatment failure.

Clinically, these results support an individualized treatment strategy. NIV may remain appropriate in selected patients with preserved mental status, manageable work of breathing, close monitoring, and early improvement in oxygenation. However, persistent severe hypoxemia, increasing respiratory effort, worsening gas exchange, intolerance, or lack of early response should prompt reassessment and consideration of invasive ventilation. The findings also reinforce the importance of collecting respiratory mechanics and time-to-treatment variables in future ICU studies, as these parameters are essential to distinguish NIV success from NIV failure and to clarify the role of delayed intubation.

### Strengths

This study has several strengths. First, it reflects real-world ICU practice during two distinct phases of the COVID-19 pandemic, allowing evaluation of outcomes before and after institutional approval of NIV use. Second, it included consecutive critically ill patients with confirmed COVID-19-related acute respiratory failure, reducing selective inclusion of favorable cases. Third, the analysis incorporated clinically relevant covariates, including severity of illness, inflammatory and coagulation markers, oxygenation status, tracheostomy, and duration of mechanical ventilation. Fourth, the use of Cox proportional hazards regression allowed time-to-event evaluation of ICU mortality. Fifth, multiple imputations and sensitivity analyses were performed to address missing data and evaluate parameter stability. Finally, the proportional hazards assumption was formally assessed, and the study was revised according to STROBE principles to improve transparency and reporting quality.^23^

### Limitations

Several limitations must be acknowledged. First, the retrospective, single-center design precludes causal inference. NIV exposure was determined by clinical judgment and institutional policy rather than randomization; therefore, residual confounding and confounding by indication remain substantial concerns. Second, although the model adjusted for measured covariates, important clinical variables were unavailable, including respiratory rate, work of breathing, ROX index, PaCO₂, vasopressor use, awake proning, NIV interface, NIV duration, ventilatory settings over time, tolerance, adherence, tidal volume during NIV, and timing from ICU admission to NIV initiation and intubation. These omissions limit mechanistic interpretation and prevent direct assessment of NIV failure pathways. Additionally, several of these respiratory support variables are directly related to the physiological mechanisms frequently proposed to explain NIV success or failure, including changes in respiratory effort, gas exchange, and lung stress. Because variables such as respiratory rate, ROX index, PaCO_2_ evolution, work of breathing, tidal volume during NIV, and objective markers of Patient Self-Inflicted Lung Injury (P-SILI) were not systematically recorded, the present study cannot directly evaluate these mechanisms. Consequently, any physiological explanation linking NIV exposure to clinical outcomes should be considered hypothesis-generating rather than directly demonstrated by the available data.

Third, the two-period design introduces temporal confounding. Viral variants, ICU strain, staffing, sedation practices, corticosteroid use, anticoagulation, proning protocols, vaccination status, thresholds for ICU admission, and thresholds for intubation may have changed during the study period. Although admission period was included in the Cox model, not all time-varying contextual factors could be measured or adjusted for. Fourth, missingness was substantial for some covariates, particularly SAPS-3 and PaO_2_/FiO_2_ ratio. Multiple imputation and sensitivity analyses were used to mitigate this limitation, but imputation cannot fully replace prospectively collected data. Fifth, NIV was modeled as a fixed exposure rather than a time-dependent intervention, which may introduce immortal time bias and time-dependent treatment bias. Although the median time from ICU admission to intubation among patients who did not receive NIV was 0 days (IQR 0–0), suggesting limited opportunity for prolonged immortal time accumulation, some degree of time-dependent bias cannot be excluded. Furthermore, precise time-stamped data required to perform a time-dependent Cox analysis were not consistently available in the retrospective database. Finally, the high intubation rate limits the ability to evaluate NIV as a definitive intubation-avoidance strategy and suggests that the results are most applicable to severely ill ICU populations rather than to less severe patients managed outside the ICU.

### Future research

Future studies should prospectively evaluate NIV in critically ill COVID-19 and non-COVID acute hypoxemic respiratory failure using standardized criteria for NIV initiation, monitoring, failure, and intubation. Multicenter designs with time-dependent exposure modeling, propensity-based approaches, and detailed physiological data are needed to distinguish treatment effect from treatment selection. In particular, future research should record respiratory drive, tidal volume, respiratory rate, work of breathing, PaCO_2_, ROX index, interface type, awake proning, vasopressor support, and time from ICU admission to NIV initiation and intubation. These data would help identify which patient subgroups may benefit from NIV and which patients require early invasive mechanical ventilation.

## Conclusions

In this retrospective cohort of critically ill patients with COVID-19-related acute respiratory failure, implementation of NIV increased substantially following institutional approval; however, intubation rates remained high across both study periods. Patients treated during the later period presented with more severe hypoxemia at ICU admission and experienced worse crude clinical outcomes.

After adjustment for measured clinical and laboratory covariates, absence of NIV use was statistically associated with a lower adjusted hazard of ICU mortality. This finding should not be interpreted as evidence that NIV increases mortality. Rather, in this real-world cohort, NIV exposure likely functioned as a marker of greater respiratory severity, clinician treatment selection, and therapeutic escalation, consistent with substantial confounding by indication.

These findings highlight the importance of careful patient selection, close monitoring, and timely recognition of NIV failure in critically ill patients with COVID-19-related acute respiratory failure. Prospective multicenter studies are needed to clarify which patient subgroups are most likely to benefit from NIV and to determine the optimal criteria for escalation to invasive mechanical ventilation.

## Institutional review board statement

The study was conducted in accordance with the principles of the Declaration of Helsinki and was approved by the Research Ethics Committee of the Grupo Hospitalar Conceição (GHC), Porto Alegre, Brazil (approval number 4164341).

## Informed consent statement

The requirement for patient consent was waived due to the retrospective nature of the study and the use of anonymized data obtained from electronic medical records.

## Authors’ contributions

Conceptualization: JM, FXA, VMCB. Investigation: JM, FXA, GMC, CC, VMCB. Data curation: JM, FXA, GMC, VMCB. Methodology: JNCZ, EASR, EMO. Formal analysis (statistical analysis): JNCZ. Writing-original draft: CC, JNCZ, EASR, EMO. Writing-review and editing: RS, JNCZ, EASR, EMO. Supervision: FXA, VMCB, JNCZ, and EASR. All authors contributed to the interpretation of the data, critically revised the manuscript, approved the final version for publication, and agreed to be accountable for all aspects of the work.

## Funding

This research received no external funding.

## Conflicts of interest

The authors declare no conflicts of interest.

## Data Availability

The data supporting the findings of this study are available in the article and its Supplementary Material. Additional anonymized data may be available from the corresponding author upon reasonable request and with permission from the Conceição Hospital Group.
