# Supplementary material for: Noninvasive ventilation use and ICU mortality in COVID-19-related acute respiratory failure: a retrospective cohort study
Source: Clinics (Sao Paulo). 2026 Sep 23;81:101159. doi: 10.1016/j.clinsp.2026.101159 (PMC13634068; doi:10.1016/j.clinsp.2026.101159)
Supplement: Supplementary file 1 [file mmc1.docx]

**Supplementary material**

**Supplementary material. Table 1.** Missing data analysis.

|  | **Total missing** | **Missing percentage** |
| --- | --- | --- |
| SAPS-3 at admission | 173 | 58.45 |
| Comorbidities | 71 | 23.99 |
| PaO_2_/FiO_2_ ratio at admission (mmHg) | 65 | 21.96 |
| D-Dimers at admission (μg/L) | 25 | 8.45 |
| Body Mass Index | 19 | 6.42 |
| C-Reactive protein at admission (mg/L) | 15 | 5.07 |
| Mechanical ventilation (days) | 5 | 1.69 |
| Period | 1 | 0.34 |
| NIV use | 0 | 0.00 |
| Tracheostomy | 0 | 0.00 |
| Gender | 0 | 0.00 |
| Age | 0 | 0.00 |
| Intubation need | 0 | 0.00 |
| ICU death | 0 | 0.00 |
| Days in ICU | 0 | 0.00 |

ICU: Intensive Care Unit; NIV: Non-invasive mechanical ventilation; SAPS-3: Simplified Acute Physiology Score-3. In red variables with more than 5% missing data.

**Supplementary material. Figure 1:** Markov Chain Monte-Carlo imputation convergence trace plots.

**Supplementary material. Table 2.** Cox model assesment.

|  | **Sensitivity analysis** | |  |
| --- | --- | --- | --- |
|  | **Naïve data (^a^p value)** | **Imputed data (^a^p value)** | **Schoenfeld´s test (^a^p value)** |
| Period (Second period) | 0.422 | 0.107 | 0.288 |
| ***NIV use (No)*** | 0.034 | 0.011 | 0.564 |
| SAPS-3 at admission | 0.219 | < 0.001 | 0.771 |
| C-Reactive protein at admission (mg/L) | 0.22 | 0.676 | 0.263 |
| D-Dimers at admission (μg/L) | 0.822 | 0.509 | 0.821 |
| PaO_2_/FiO_2_ ratio at admission (mmHg) | 0.064 | 0.02 | 0.328 |
| Body Mass Index | 0.358 | 0.375 | 0.292 |
| Tracheostomy (Yes) | 0.895 | 0.026 | 0.073 |
| Mechanical ventilation (days) | 0.132 | < 0.001 | 0.001 |
| Overall |  |  | 0.096 |

Green: Robust variables in sensitivity analysis. ICU: Intensive Care Unit; NIV: Non-invasive mechanical ventilation; SAPS-3: Simplified Acute Physiology Score-3. ^a^Statistically significant if p < 0.05 (shown in red).
